# A Diagnostic Trap: Subhepatic Appendicitis Mimicking Hepatobiliary Disorders

**DOI:** 10.7759/cureus.96798

**Published:** 2025-11-13

**Authors:** Adhya M Tom, Sofia S Ali, Jagat S Gopinath, Chris M Prince, Haris K Punnackal

**Affiliations:** 1 Emergency Medicine, Gulf Medical University, Ajman, ARE; 2 Emergency Medicine, Georgian National University SEU, Tbilsi, GEO; 3 Emergency Medicine, Dubai Hospital, Dubai, ARE

**Keywords:** computed tomography abdomen, hepatobiliary anatomy, laproscopic appendectomy, subhepatic appendicitis, upper abdominal pain

## Abstract

Subhepatic appendicitis is a rare anatomical variant of acute appendicitis that can mimic hepatobiliary or pancreatic pathology, often resulting in diagnostic delays and increased morbidity. We report the case of a 39-year-old woman who presented with acute upper abdominal pain and vomiting, without fever, right lower quadrant tenderness, and other gastrointestinal symptoms. Laboratory findings showed leukocytosis with neutrophilia and lymphopenia, while liver and renal function tests were within normal limits. Abdominal ultrasound was unremarkable; however, contrast-enhanced computed tomography (CT) revealed an acutely inflamed subhepatic appendix. The patient underwent a laparoscopic appendectomy, which confirmed early inflammatory mass formation and full peritoneal coverage of the appendix. Careful dissection of the peritoneum and mesoappendix with cauterization of the appendicular artery was performed successfully. Postoperative recovery was uneventful, and the patient was discharged the following day. This case highlights the diagnostic challenge of subhepatic appendicitis and underscores the pivotal role of CT imaging in identifying atypical presentations. Laparoscopic appendectomy remains a safe and effective therapeutic option, offering rapid recovery and favorable outcomes.

## Introduction

Appendicitis refers to inflammation of the appendix and, if not managed promptly, may progress to complications such as abscess formation, bowel obstruction, peritonitis, or even mortality [1]. It is one of the most frequent surgical emergencies, affecting around 250,000 individuals annually in the United States and accounting for nearly one million hospital bed days [2].

The classic clinical picture typically begins with periumbilical pain that later shifts to the right iliac fossa, accompanied by anorexia, fever, and localized tenderness with guarding [3]. On abdominal examination, focal tenderness and muscle rigidity are usually evident once the pain localizes. Laboratory findings often demonstrate leukocytosis with a neutrophilic predominance, while C-reactive protein levels are commonly elevated [4]. Approximately 90% of patients treated with antibiotics are able to avoid surgery during the initial admission. The other 10% that fail to respond to antibiotics require an emergency appendectomy. Recurrence rates of nonoperated patients within one year are as high as 20-30% [5].

Subhepatic appendicitis is an exceedingly rare presentation, accounting for 0.01% of acute appendicitis cases [6]. It typically arises from intestinal malrotation or failure of the cecum to descend properly during embryological development [7]. Because of its atypical location, subhepatic appendicitis frequently presents with features similar to liver or biliary tract disease. This overlap can delay recognition, sometimes resulting in unwarranted cholecystectomy, recurrent hospital visits, greater morbidity, and additional healthcare expenses [8]. 

This case is significant in the context of the United Arab Emirates, where documentation of rare appendiceal variants remains limited. Presenting such an uncommon entity emphasizes the need for heightened clinical suspicion of atypical appendicitis presentations in the region. By adding to the regional body of literature, this report may support earlier recognition, reduce diagnostic errors, and ultimately improve surgical decision-making and patient outcomes.

## Case presentation

A 39-year-old Saudi woman presented to the emergency department with complaints of upper abdominal pain since morning, associated with vomiting. She was initially evaluated at a primary health center and referred due to persistent pain. Patient reported not consuming unusual food the previous day. There was no history of lower abdominal pain, chest pain, fever, breathlessness, giddiness, leg swelling, dysuria, or altered consciousness. No other household members had similar symptoms. She denied diarrhea or loose stools. No significant past medical, surgical, or family history was documented.

On examination, the patient was alert and oriented, with stable vital signs: blood pressure of 118/72 mmHg, pulse of 72/min, temperature of 36.1°C, SpO_2_ 100% on room air, and respiratory rate of 18/min. The abdomen was soft with tenderness localized to the right upper quadrant and epigastric region. No additional issues were noted in the other systems.

The patient’s laboratory results revealed a white blood cell count of 15.2 × 10^3^/µL, with an elevated absolute neutrophil count of 14.2 × 10^3^/µL. Lymphopenia was noted, with an absolute lymphocyte count of 0.7 × 10^3^/µL. Hemoglobin was 12.5 g/dL, platelet count was 264 × 10^3^/µL, C-reactive protein measured 2.4 mg/L, and serum creatinine was 0.66 mg/dL. All other laboratory parameters were within normal limits (Table 1).

**Table 1:** Lab values of patient with subhepatic appendicitis

Lab Tests	Lab Values	Reference Range
White blood cells	15.2 × 10³/µL	4.0-11.0 × 10³/µL
Neutrophil	14.2 × 10³/µL	1.5-8.0 × 10³/µL
Absolute lymphocyte count	0.7 × 10³/µL	1.0-3.0 × 10³/µL
Hemoglobin	12.5 g/dL	12.0-15.5 g/dL
Platelet count	264 × 10³/µL	150-450 × 10³/µL
C-reactive protein	2.4 mg/L	<5 mg/L
Serum creatinine	0.66 mg/dL	0.6-1.1 mg/dL

**Figure 1:**
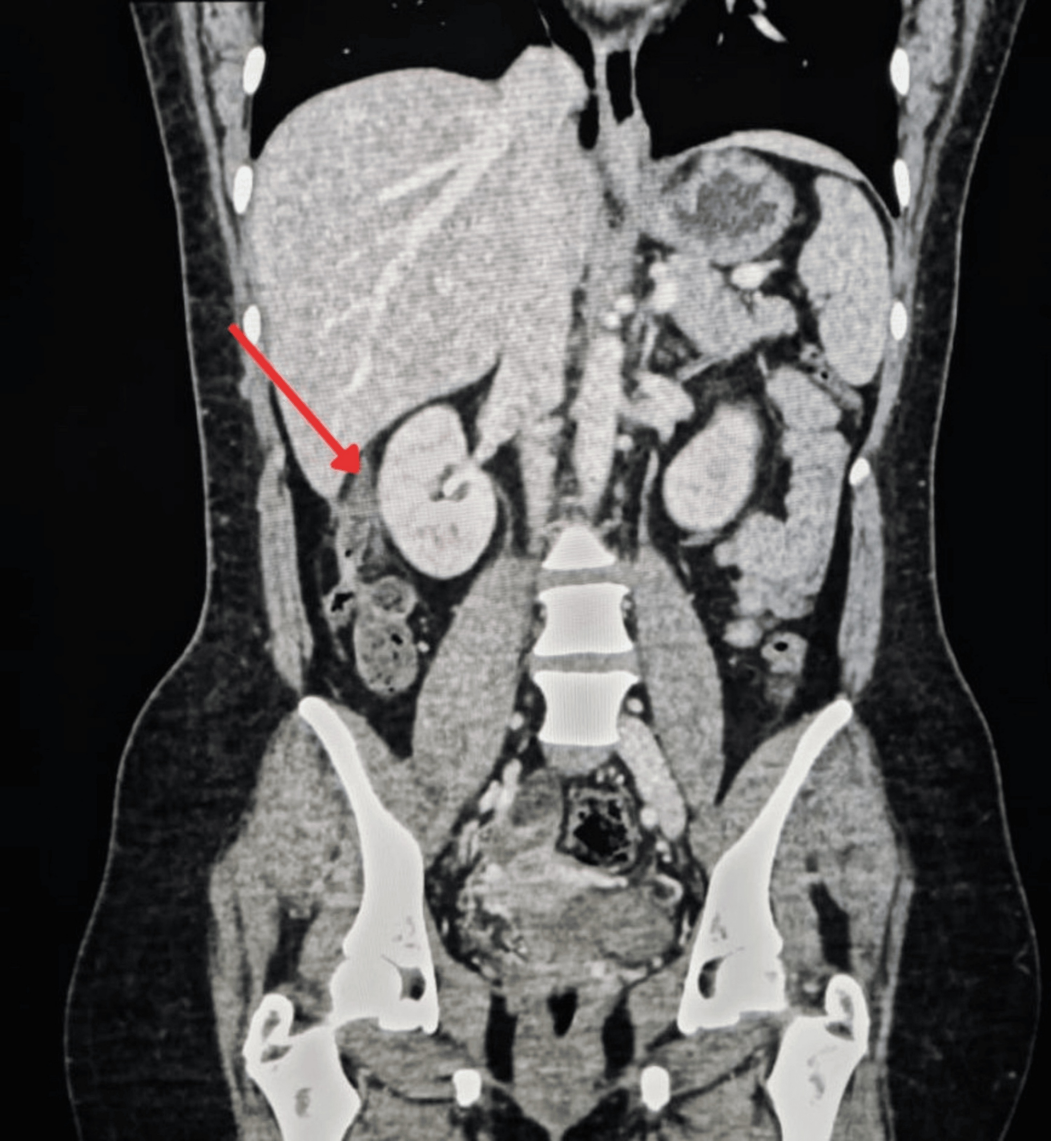
Computed tomographic image showing subhepatic appendicitis (arrow)

Abdominal ultrasound demonstrated no abnormalities. The liver, gallbladder, pancreas, kidneys, and spleen were clearly visualized and appeared normal, with no evidence of ascites. Given the inconclusive findings, a contrast-enhanced computed tomography (CT) scan was performed. CT imaging revealed an acutely inflamed subhepatic appendix (Figure 1).

The patient underwent a laparoscopic appendectomy. Intraoperatively, the appendix was identified in the subhepatic region, demonstrating early mass formation and being completely covered by peritoneum. Dissection of the peritoneum was performed using a harmonic scalpel (Ethicon Endosurgery LLC, USA), followed by division of the mesoappendix and cauterization of the appendicular artery. The procedure was uneventful, and the patient showed excellent postoperative recovery, being mobilized on the first day and discharged the following day.

## Discussion

This case highlights a rare presentation of subhepatic appendicitis, a condition often misdiagnosed due to its atypical anatomical location. The patient, a 39-year-old woman, presented with upper abdominal pain and vomiting, initially raising suspicion for hepatobiliary or pancreatic pathology. The absence of classic right lower quadrant pain, fever, or gastrointestinal disturbances complicated early diagnosis, reflecting the diagnostic challenge posed by abnormally positioned appendices. Subhepatic appendicitis is exceedingly uncommon, accounting for approximately 0.08% of all appendicitis cases [9]. Previous reports similarly indicate that patients often present with predominant right upper quadrant pain, frequently leading to misdiagnosis as biliary pathology [10,11].

Laboratory investigations demonstrated leukocytosis with neutrophilia and lymphopenia, consistent with an acute inflammatory process, though nonspecific. Other laboratory parameters, including liver and renal function tests, were unremarkable, potentially diverting clinical suspicion away from a surgical etiology.

Imaging played a crucial role in establishing the diagnosis. Initial abdominal ultrasound failed to reveal abnormalities, reflecting its known limitations in detecting atypically located appendices. In contrast, contrast-enhanced CT provided clear visualization of the inflamed subhepatic appendix invaginated beneath the peritoneum, supporting the position of CT as the gold standard for atypical appendicitis. While ultrasound has poor sensitivity for unusual appendiceal locations, CT demonstrates high diagnostic performance, with reported sensitivity of 100%, specificity of 95%, and overall accuracy of 98% [12]. In cases where CT is inconclusive and clinical suspicion persists, diagnostic laparoscopy remains a valuable tool [13].

The patient underwent a successful laparoscopic appendectomy. Intraoperative findings confirmed early mass formation with complete peritoneal coverage, requiring meticulous dissection of the peritoneum and mesoappendix. The patient’s rapid postoperative recovery and early discharge underscore the advantages of minimally invasive surgery, even in rare anatomical presentations. Laparoscopy is particularly beneficial in clinically stable patients without generalized peritonitis, offering both diagnostic and therapeutic utility [14]. Although laparoscopic subhepatic appendectomy is safe and effective, the procedure can be technically challenging due to the appendix’s unusual location, and the incidence of complicated appendicitis is higher in these cases [15].

## Conclusions

Because of its unusual anatomical position, subhepatic appendicitis is an uncommon and sometimes misdiagnosed form of acute appendicitis. This example demonstrates the difficulties in making an early diagnosis, particularly when there are no traditional symptoms and nonspecific laboratory results. Laparoscopic appendectomy provided both therapeutic and diagnostic advantages, whereas contrast-enhanced CT was crucial in detecting the inflamed appendix. Even with uncommon appearances, excellent results can be achieved with early detection and minimally invasive surgical intervention. When patients experience localized upper abdominal pain, clinicians should rule out atypical appendicitis to prevent unnecessary treatments, delayed diagnosis, and related morbidity.
